# Níveis Reduzidos de CTRP3 em Pacientes com Doença Arterial Coronariana Estável Relacionados à Presença de Fibrilação Atrial Paroxística

**DOI:** 10.36660/abc.20200669

**Published:** 2021-11-17

**Authors:** Arafat Yildirim, Mehmet Kucukosmanoglu, Hilmi Erdem Sumbul, Mevlut Koc

**Affiliations:** 1 Departamento de Cardiologia University of Health Sciences - Adana Health Practice and Research Center Adana Turquia Departamento de Cardiologia , University of Health Sciences - Adana Health Practice and Research Center , Adana - Turquia; 2 Departamento de Medicina Interna University of Health Sciences - Adana Health Practice and Research Center Adana Turquia Departamento de Medicina Interna , University of Health Sciences - Adana Health Practice and Research Center , Adana - Turquia

**Keywords:** Adipocinas, Proteína 3 Induzida por Fator de Necrose Tumoral Alfa, Doença da Artéria Coronariana, Fibrilação Atrial, Fatores de Risco, Arritmia Cardíaca

## Abstract

**Fundamento:**

Os níveis de Proteína 3 relacionada ao fator de necrose tumoral/complemento sérico C1q (CTRP3) e a relação com a fibrilação atrial (FA) na doença arterial coronária estável (DAC) não estão claros atualmente.

**Objetivos:**

O objetivo deste estudo foi investigar a mudança nos níveis séricos de CTRP3 e sua relação com a FA paroxística em DAC estável.

**Método:**

O estudo incluiu 252 pacientes com DAC e 50 controles saudáveis com idade/sexo compatíveis. Os níveis séricos de CTRP3 foram medidos, além da anamnese de rotina, exame físico, exames laboratoriais e ecocardiograma. Os pacientes foram divididos em grupos com e sem DAC e indivíduos com DAC com e sem FA paroxística. Os valores eram estatisticamente significativos quando p<0,05.

**Resultados:**

Os níveis séricos de CTRP3 foram significativamente menores em pacientes com DAC do que no grupo controle (p<0,001). A FA foi detectada em 28 pacientes (15,08%) no grupo DAC. A frequência de hipertensão e do sexo feminino, a proteína C reativa de alta sensibilidade (PCR-as), o nitrogênio ureico no sangue, os níveis de creatinina e o diâmetro diastólico do átrio esquerdo foram maiores (p<0,05 para cada um), e os níveis de CTRP3 foram mais baixos em pacientes com FA (p<0,001). Na análise de regressão logística, os níveis séricos de CTRP3 e os diâmetros diastólicos do átrio esquerdo foram independentemente determinados pelos pacientes com FA (p<0,01 para cada um). Nesta análise, observamos que cada 1 ng/mL de redução nos níveis de CTRP3 aumentou o risco de FA em 10,7%. Na análise ROC dos valores de CTRP3 para detectar pacientes com FA, a área da curva ROC para CTRP3 foi 0,971 (0,951–991) e considerada estatisticamente significativa (p<0,001). Quando o ponto de corte de CTRP3 foi considerado em 300 ng/mL, demonstrava a presença de FA com 87,9% de sensibilidade e 86,8% de especificidade.

**Conclusão:**

Os níveis séricos de CTRP3 caíram significativamente em pacientes com DAC estável, e níveis reduzidos de CTRP3 estiveram relacionados à presença de FA paroxística nesses pacientes.

## Introdução

As adipocinas são polipeptídeos secretados do tecido adiposo, essenciais para regular o metabolismo energético.^1^ A proteína 3 relacionada ao fator de necrose tumoral/complemento sérico C1q (CTRP3) é um novo membro da família das adipocinas. Os principais efeitos da CTRP3 são inibição do estresse oxidativo induzido pela glicose alta, anti-inflamatório, inibição da apoptose e da fibrose, promoção da angiogênese e inibição da gliconeogênese.^2 - 5^ A CTRP3, que foi investigada por seus efeitos metabólicos, também demonstrou reduzir a incidência de doenças cardiovasculares (CV).^6 - 9^

A Doença Arterial Coronariana (DAC) é a doença cardiovascular mais comum, enquanto a fibrilação atrial (FA) é a arritmia cardíaca mais comum. A arritmia mais importante e frequente em pacientes com DAC é a FA. Ambas as doenças apresentam fatores de risco como hipertensão (HT), diabetes mellitus (DM), apneia do sono, obesidade e o hábito de fumar.^10^

Os níveis de CTRP3 foram investigados em pacientes com síndrome coronariana aguda (SCA) e angina estável.^11^ Neste estudo, os níveis séricos de CTRP3 foram reduzidos em pacientes com SCA e angina estável, e esses resultados sugerem que a CTRP3 pode ser útil para avaliar o risco de DAC.^11^ A fisiopatologia e o mecanismo da FA em pacientes com DAC são complexos e multifatoriais. Porém, a fibrose atrial induzida pelo estiramento, o aumento da inflamação, o remodelamento vascular, a apoptose e o estresse oxidativo, a hipocontratilidade, a infiltração de gordura, a isquemia, a disfunção dos canais iônicos e a instabilidade do Ca2+ estão relacionados à presença e à ocorrência de FA.^12^ O fato de que níveis mais baixos de CTRP3 estão relacionados à inflamação, fibrose, apoptose e estresse oxidativo nos levou à hipótese de que a FA, que é a arritmia mais comum em pacientes com DAC, pode estar associada a níveis reduzidos de CTRP3. Até onde sabemos, não há estudos que investigam a relação entre os níveis séricos de CTRP3 e FA em pacientes com DAC estável.

Assim, investigamos as mudanças nos níveis séricos de CTRP3 em pacientes com DAC estável e a relação entre os níveis de CTRP3 e a presença de FA paroxística nesses pacientes.

## Materiais e Métodos

### População do Estudo

Este estudo transversal incluiu 252 pacientes com DAC estável, internados na clínica de arritmia do nosso hospital, cujas idades e sexos eram similares aos 50 controles saudáveis. Os pacientes foram avaliados por dois cardiologistas para a presença de FA utilizando um holter de 72 horas (eletrocardiografia – ECG) antes de serem incluídos no estudo. Pelo menos 30 segundos de duração da FA são necessários para diagnosticar a FA.^12^ De acordo com estudos prévios comparando as médias de CTRP3 entre os grupos com DAC e controle com o teste-t para duas amostras, com 5% de significância e poder de 80%, 50 pacientes são necessários para cada um dos dois grupos. Com base no conhecimento de que 17-47% de pacientes com DAC têm FA,^10^ no começo do estudo 100 pacientes com DAC estavam planejados para se inscrever na pesquisa para que pudéssemos analisar pelo menos 30 deles com FA. Porém, durante o processo, o número de pacientes com DAC e FA foi muito menor do que o esperado. Assim, o estudo foi realizado com 252 indivíduos com DAC. Pacientes com SCA, com uso de medicação para arritmia, insuficiência cardíaca com fração de ejeção reduzida, aqueles com histórico de doença hepática, insuficiência renal grave, doença valvular cardíaca moderada a grave, doença da tireoide ativa, suspeita de câncer e/ou gravidez, e aqueles que não quiserem ser incluídos no estudo, foram excluídos. O estudo foi realizado de acordo com as recomendações da Declaração de Helsinki para pesquisa médica envolvendo humanos, e o protocolo foi aprovado pelo Comitê de Ética da instituição. Os termos de consentimento foram explicados detalhadamente aos pacientes, que foram incluídos na pesquisa após assinarem o documento.

Um histórico detalhado foi analisado e exames foram realizados em todos os indivíduos. Em seguida, as características demográficas dos grupos foram coletadas; idade, sexo, histórico de hipertensão, diabetes mellitus, fato de ser fumante e hiperlipidemia. O índice de massa corporal (IMC) foi calculado ao medir peso e altura. Os parâmetros laboratoriais, como proteína C reativa de alta sensibilidade (PRC-as), glicose, funções renais e hepáticas, porção N-terminal do pró-hormônio do peptídeo natriurético do tipo B (NT-proBNP) e valores de lipídeos em todos os pacientes e controles foram medidos.

Os tratamentos em andamento foram continuados antes de os pacientes serem examinados com o holter de 72 horas. Os indivíduos foram instruídos a seguir com suas rotinas normais. Um monitor holter programado para 72 horas, com gravação automática e acompanhamento do ritmo, foi utilizado (GE Seer 1000, EUA). Os pacientes foram para casa após receber instruções sobre o equipamento. Depois de 72 horas, o monitor foi removido e as gravações foram revisadas e concluídas com o software Mars V8 ambulatory ECG. Informações detalhadas foram dadas aos pacientes. Os resultados do holter foram avaliados por dois eletrofisiologistas (AY e MK) para diagnosticar a FA silenciosa, que não sabiam que os fatores de risco e dados clínicos, assim como os pacientes em si, seriam incluídos no estudo. Como resultado desta avaliação, se houvesse algum resultado inconsistente entre os dois eletrofisiologistas (inter-observador), a decisão definitiva era tomada por um terceiro eletrofisiologista de nossa clínica (MK). No holter de 12 derivações: ı) atividade atrial irregular e variabilidade na duração do ciclo atrial (menor que 200 ms); ıı) irregularidade no intervalo R-R; ııı) ausência de onda P significativa recorrente; ıv) em vez de ondas P, formas e tamanhos irregulares e diferentes de ondas de fibrilação podem ser vistos; v) por fim, frequências ventriculares irregulares e variadas podem ser considerada como FA.

Amostras de sangue para os níveis de CTRP foram coletadas dos grupos de pacientes e controles às 8h da manhã, após um período de 12 horas de jejum. As amostras de sangue venoso foram coletadas e centrifugadas a 4000 rpm por pelo menos 10 minutos. Obtivemos amostras séricas, que foram armazenadas a -80ºC até a análise. Para medir os níveis séricos de CTRP3, foram utilizados kits ELISA (do inglês, *Enzyme-Linked Immunosorbent Assay* ) (Adipogen, Coreia do Sul, Cat# AG-45A0042EK-KI01). A sensibilidade da CTRP3 estava dentro do intervalo do estudo: 1 ng/mL – 1000 ng/mL, e os resultados foram demonstrados em ng/mL.

Todos os exames ecocardiográficos foram realizados no EPIQ 7 (Philips Healthcare, Andover, Massachusetts, EUA). As diretrizes da Sociedade Americana de Ecocardiografia foram utilizadas para obter imagens. Quando os pacientes eram monitorados do lado esquerdo, o eixo longo e curto paraesternal foi obtido, assim como cortes apical das câmaras 5, 4 e 2, e pelo menos 3 ciclos consecutivos.^13^

O exame do eixo longo paraesternal (modo M) demonstrou o diâmetro diastólico do átrio esquerdo (LAd). Os volumes diastólico e sistólico do ventrículo esquerdo (VEd e VEs) e a fração de ejeção do ventrículo esquerdo (FEVE) foram calculados em uma ecocardiografia utilizando o método de Simpson no corte apical das câmaras 4 e 2.^14^

### Análise estatística

Todas as análises foram realizadas com o software SPSS 22.0 (Chicago, IL, EUA). O teste de Kolmogorov-Smirnov foi utilizado para avaliar a distribuição das variáveis contínuas, que foram expressas como média ± desvio padrão, ou mediana – intervalo interquartil. As variáveis categóricas foram expressas em números e porcentagens. As variáveis contínuas que tiveram distribuição normal foram comparadas utilizando o teste t de Student não pareado, enquanto o teste U de Mann-Whitney foi adotado para comparar as diferenças entre os dois grupos independentes quando a variável dependente era ou ordinal ou contínua, mas com distribuição normal. O teste de qui-quadrado foi usado para comparar as variáveis categóricas. Uma análise de regressão logística foi realizada para determinar os marcadores independentes entre os pacientes com FA. Uma análise da curva ROC foi feita para reavaliar os níveis de CTRP3 para detectar pacientes com FA, e para determinar o valor limite da CTRP3. O valor da área dentro da curva foi utilizado para medir a precisão do teste. A significância estatística aceita foi de p <0,05.

## Resultados

Os dados do estudo foram comparados em dois grupos: pacientes com DAC estável e o grupo controle. Além disso, pacientes com DAC foram agrupados e comparados como pacientes com e sem FA paroxística. Do total dos 302 indivíduos, 252 pacientes com DAC estável (sexo feminino, n=85, 33,7%; média de idade 61,6 ± 11,4) e 50 controles (sexo feminino, n=20, 40,0%; média de idade: 60,4 ± 9,2) foram incluídos no estudo. Dos pacientes com DAC incluídos no estudo, 161 (63,9%) tinham hipertensão, 84 (33,3%) tinham diabetes mellitus, 86 (34,1%) tinham hiperlipidemia e 69 (27,4%) eram fumantes. A FA foi detectada em 38 (15,07%) pacientes com DAC estável.

As características demográficas e achados laboratoriais de todos os participantes estão resumidos na Tabela 1 . Quando as características dos pacientes e dos controles foram comparadas, a idade e o sexo foram similares (61,6 ± 11,4 vs. 60,4 ± 9,2; p=0,514 e 33,7% vs. 40,0%, p=0,224, respectivamente). Ao serem comparados com o grupo controle, os níveis de glicose sérica, colesterol total e LDL foram significativamente mais altos em pacientes com DAC estável (146 ± 75 vs. 95 ±12; p<0,001, 184 ± 42 vs. 152 ± 36; p=0,012, 120 ± 34 vs. 100 ± 25; p=0,020, respectivamente). Os níveis séricos de CTRP3 foram estatisticamente mais baixos, enquanto os níveis de PCR-as foram maiores em pacientes com DAC estável [331 ± 46 vs. 432 ± 46; p<0,001 e 1,09 (0,95 – 1,31), 0,89 (0,76 – 1,01); p=0,030, respectivamente]. Outros dados laboratoriais foram similares entre os grupos. Quando os parâmetros da ecocardiografia foram comparados, as dimensões do diâmetro diastólico do átrio esquerdo foram significativamente maiores (43 ± 4,6 vs. 38 ± 4,1; p=0.012), e a FEVE foi menor (52 ± 6,9 vs. 61 ± 4,5; p=0,019) em pacientes com DAC estável.

**Tabela 1:** – Achados demográficos e laboratoriais de pacientes com doença arterial coronariana e controles saudáveis

Variável	Pacientes com DAC n=252	Controles saudáveis n=50	p
Idade (anos)	61,6 ± 11,4	60,4 ± 9,2	0,514
Gênero (feminino), n (%)	85 (33,7)	20 (40,0)	0,224
IMC (kg/m^2^)	25,4 ± ,.23	25,9 ± 1,30	0,122
Glicose (mg/dL)	146 ± 75	95 ± 12	<0,001
Nitrogênio ureico no sangue (mg/dL)	36,2 ± 15,6	33,1 ± 14,5	0,192
Creatinina (mg/dL)	0,90 (0,75 – 0,99)	0,80 (0,70 – 0,92)	0,334
Colesterol total (mg/dL)	184 ± 42	152 ± 36	**0,012**
LDL (mg/dL)	120 ± 34	100 ± 25	**0,020**
HDL (mg/dL)	40 ± 12	42 ± 9,8	0,856
Triglicérides (mg/dL)	149 (130 – 175)	151 (125 – 186)	0,956
PCR-as (mg/dL)	1,09 (0,95 – 1,31)	0,89 (0,76 – 1,01)	**0,030**
CTRP3 (ng/mL)	331 ± 46	432 ± 46	**<0,001**
Volume VEd (mL)	102 ± 19	95 ± 11	0,102
Volume VEs (mL)	46 ± 14	41 ± 10	0,112
Diâmetro diastólico do átrio esquerdo (mm)	43 ± 4.6	38 ± 4,1	**0,012**
FEVE (%)	52 ± 6,9	61 ± 4,5	**0,019**

*Os valores foram demonstrados como média ± desvio padrão, mediana – intervalo interquartil ou n (%), IMC: índice de massa corporal; DAC: doença arterial coronariana; CTRP3: proteína 3 relacionada ao fator de necrose tumoral/complemento sérico C1q; HDL: lipoproteína de alta densidade; PCR-as: proteína C reativa de alta sensibilidade; LDL: lipoproteína de baixa densidade; FEVE: fração de ejeção do ventrículo esquerdo; VEd: volume diastólico do ventrículo esquerdo; VEs: volume sistólico do ventrículo esquerdo.*

As características demográficas e os achados laboratoriais de pacientes com DAC estável, de acordo com a presença de FA, estão resumidos na Tabela 2 . Os dados demográficos dos pacientes com e sem FA paroxística, frequência do sexo feminino, idade e hipertensão foram estatisticamente mais altos em pacientes com FA (50,0% vs. 30,8%; p= 0,019, 66,8 ± 10,7 vs. 60,3 ± 11,2; p=0,001, 78,9% vs. 61,2%; p=0,025, respectivamente). Quando comparados com pacientes com DAC estável sem FA paroxística, os níveis de nitrogênio ureico no sangue e creatinina [44,5 ± 21,9 vs. 34,8 ± 13,8; p= 0,012, 1,20 (0,90 – 1,45) vs. 0,85 (0,70 – 1,35); p=0,004, respectivamente] foram significativamente mais altos em pacientes com DAC estável e FA paroxística. Além disso, os níveis séricos de CTRP3 foram estatisticamente mais baixos, enquanto os níveis de PCR-as foram mais altos em pacientes com DAC estável e FA paroxística [262 ± 27 vs. 343 ± 38; p=<0,001, 3,15 (2,22 – 3,95) vs. 0,80 (0,65 -1,02); p<0,001, respectivamente]. Outros dados laboratoriais foram similares entre os dois grupos. Quando os parâmetros da ecocardiografia foram comparados, o diâmetro diastólico do átrio esquerdo foi significativamente maior (46 ± 5,4 vs. 41 ± 4,2; p=0,002) e a FEVE foi significativamente menor (49 ± 4,9 vs. 53 ± 6,1; p=0,049) em pacientes com DAC estável e FA paroxística.

**Tabela 2:** – Achados demográficos e laboratorais da DAC em pacientes de acordo com a presença da fibrilação atrial

Variável	Fibrilação atrial paroxística (+) n=38	Fibrilação atrial paroxística (-) n=214	p
Idade (Anos)	66,8 ± 10,7	60,3 ± 11,2	**0,001**
Gênero (feminino), n (%)	19 (50,0)	66 (30,8)	**0,019**
Hipertensão, n (%)	30 (78,9)	131 (61,2)	**0,025**
Diabetes mellitus, n (%)	15 (39,5)	69 (32,2)	0,245
Fumante ativo, n (%)	4 (10,5)	65 (30,4)	0,224
Hiperlipidemia, n (%)	13 (34,2)	73 (34,1)	0,224
IMC (kg/m^2^)	25,3 ± 2,18	25,5 ± 2,24	0,576
Glicose (mg/dL)	160 ± 65	144 ± 78	0,207
Nitrogênio ureico no sangue (mg/dL)	44,5 ± 21,9	34,8 ± 13,8	0,012
Creatinina (mg/dL)	1,20 (0,90 – 1,45)	0,85 (0,70 – 1,35)	**0,004**
Colesterol total (mg/dL)	179 ± 42	184 ± 42	0,518
LDL (mg/dL)	116 ± 33	121± 34	0,380
HDL (mg/dL)	41 ± 13	40 ± 12	0,823
Triglicérides (mg/dL)	140 (115 – 165)	150 (110 -182)	0,663
PCR-as (mg/dL)	3,15 (2,22 – 3,95)	0,80 (0,65 -1,02)	**<0,001**
CTRP3 (ng/mL)	262 ± 27	343 ± 38	**<0,001**
Volume VEd (mL)	104 ± 22	96 ± 18	0,305
Volume VEs (mL)	52 ± 15	48 ± 11	0,456
Diâmetro diastólico do átrio esquerdo (mm)	46 ± 5,4	41 ± 4,2	**0,002**
FEVE (%)	49 ± 4,9	53 ± 6,1	**0,049**

*Os valores foram demonstrados como média ± desvio padrão, mediana – intervalo interquartil ou n (%); IMC: índice de massa corporal; DAC: doença arterial coronariana; CTRP3: proteína 3 relacionada ao fator de necrose tumoral/complemento sérico C1q; HDL: lipoproteína de alta densidade; PCR-as: proteína C reativa de alta sensibilidade; LDL: lipoproteína de baixa densidade; FEVE: fração de ejeção do ventrículo esquerdo; VEd: volume diastólico do ventrículo esquerdo; VEs: volume sistólico do ventrículo esquerdo.*

Parâmetros demográficos, clínicos e laboratoriais associados à FA paroxística na análise univariada foram avaliados pela regressão logística multivariada. Os níveis séricos de CTRP3 (OR: 0,893; IC95%, 0,856–0,931; p<0,001) e os valores do diâmetro diastólico do átrio esquerdo (OR: 1,160; IC95%, 1,101–1,229; p=0,003) determinaram, de forma independente, os pacientes com FA paroxística ( Tabela 3 ). Nesta análise, cada 1 ng/mL a menos no nível de CTRP3 aumentou as chances de se ter FA paroxística em 10,7%.

**Tabela 3:** – Análise de regressão variável para detector DAC em pacientes com fibrilação atrial

Variável	OR	IC95%	p
Diâmetro diastólico do átrio esquerdo (mm)	1,160	1,101 – 1,229	0,003
CTRP3 (ng/mL)	0,893	0,856 – 0,931	<0,001

*CTRP3: proteína 3 relacionada ao fator de necrose tumoral/complemento sérico C1q; OR: odds ratio; IC: intervalo de confiança.*

**Figura 1:**
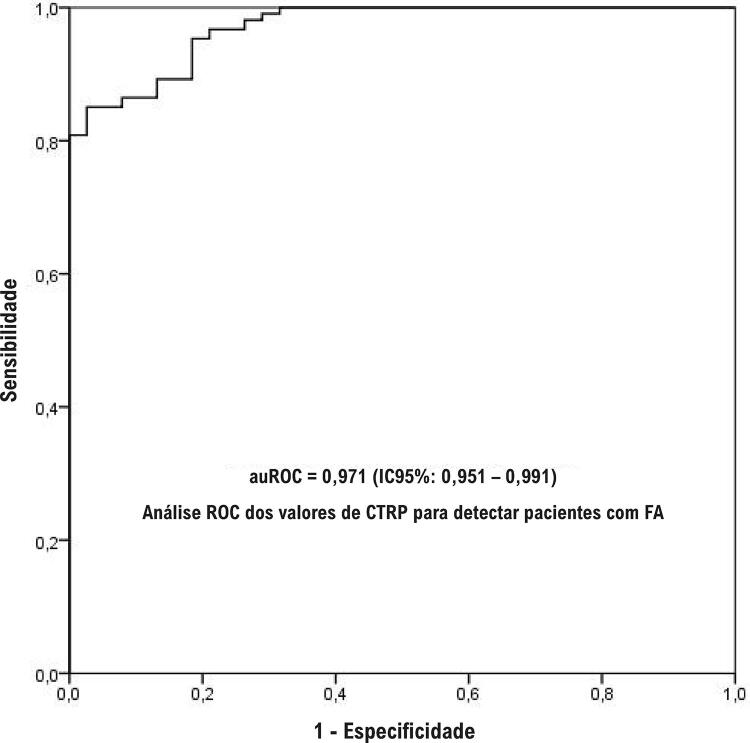
– Curva característica de operação do receptor dos valores de CTRP para determiner pacientes com FA paroxística.

Na análise da curva ROC dos valores de CTRP3 para detectar pacientes com FA paroxística, a área dentro da curva ROC para CTRP3 foi 0,971 (IC95%: 0,951–0,991), estatisticamente significativo (p<0,001 e Figura 1 ). Quando o ponto de corte da CTRP3 foi considerado em 300 ng/mL, considerou-se como fator preditivo da FA, com 87,9% de sensibilidade e 86,8% de especificidade.

## Discussão

O achado mais importante do nosso estudo é o de que os níveis séricos de CTRP3 foram significativamente mais baixos em pacientes com DAC estável quando comparados aos controles. Níveis reduzidos de CTRP3 estiveram relacionados à FA paroxística, que é a forma mais comum de arritmia em pacientes com DAC estável. Na literatura, somente um estudo reportou que os níveis séricos de CTRP3 diminuíram em pacientes com angina estável e SCA; porém, nosso estudo trata da importância dos níveis séricos de CTRP3 para prever a FA que pode se apresentar nesses pacientes. Quando o valor limite para o nível sérico de CTRP3 é considerado como 300 ng/mL, prevê o risco de desenvolver a FA paroxística com sensibilidade e especificidade aceitáveis. Por isso, nosso estudo traz dados importantes para a literatura.

Nos estudos sobre os efeitos da CTRP3 no sistema cardiovascular mostra-se que: i) reduz o estresse oxidativo, ii) inibe a apoptose, iii) tem efeitos anti-inflamatórios e antiaterogênicos, iv) reduz o desenvolvimento de fibrose, v) inibe a gliconeogênese e, como resultado de todos esses efeitos, a CTRP3 reduz as chances de desenvolver doenças cardiovasculares (CV).^2^ Considerando seus efeitos atuais, observou-se que a CTRP3 pode, em termos fisiopatológicos, proteger e melhorar os aspectos clínicos de pacientes com DAC. Níveis séricos de CTRP3 foram avaliados em pacientes com insuficiência cardíaca com fração de ejeção reduzida (ICFEr), SCA, angina estável e dissecção aórtica aguda; e os níveis sérios de CTRP3 foram significativamente menores.^6 , 11 , 17^ Em estudos *in vivo* em ratos, a CTRP3 demonstrou melhorar as funções de contração cardíaca com efeitos antiapoptóticos e proangiogênicos após uma isquemia do miocárdio.^18^ Da mesma forma, em pacientes com SCA e angina estável, os níveis séricos de CTRP3 foram significativamente menores do que nos indivíduos do grupo controle.^11^ A CTRP3 pode ter tido esses efeitos positivos com impactos antiaterogênicos e anti-inflamatórios.^15^

A arritmia mais comum e importante em pacientes com DAC é a FA. Ambas as condições apresentam fatores de risco como hipertensão, diabetes mellitus, apneia do sono, obesidade e o hábito de fumar. A prevalência de DAC em pacientes com FA varia de 17 a 46,5%, enquanto a prevalência de FA entre pacientes com DAC é baixa, estimando-se variar entre 0,2 e 5%.^10^ Além disso, a inflamação tem papel etiológico em ambas as doenças. Não é fácil explicar a patogênese da FA em pacientes com DAC por um mecanismo. A ativação dos fibroblastos, a melhor deposição do tecido conjuntivo e a fibrose são as marcas deste processo.^12^ Também deve-se considerar que a infiltração gordurosa no átrio, infiltrados inflamatórios, hipertrofia dos miócitos, necrose e amiloidose são encontrados em pacientes com FA e condições concomitantes que predispõem a FA.

O fato de que o nível de CTRP3 tem um efeito positivo em mecanismos essenciais, que são efetivos na fisiopatologia da FA, levou-nos a considerar que pode haver uma relação entre os níveis de CTRP3 e FA. Como resultado, em nosso estudo, o nível sérico de CTRP3 foi significativamente menor no grupo de pacientes com FA devida à DAC do que no grupo de pacientes sem FA. Assim, nosso objetivo foi analisar as mudanças nos níveis séricos de CTRP3 em pacientes com DAC estável e a relação entre os níveis de CTRP3 e a presença de FA paroxística nesses pacientes.

Diversos estudos anteriores reportaram que os fatores de risco do metabolismo, como obesidade, maior circunferência da cintura, pressão arterial, glicose de jejum e resistência à insulina, estão negativamente associados aos níveis séricos de CTRP3.^19 - 21^ Um achado semelhante foi demonstrado pelo nível sérico de CTRP3 significativamente menor em pacientes com DM.^22^ A DAC e a DM estão muito relacionadas, e demonstrou-se que os níveis séricos de CTRP3 são reduzidos em ambos os grupos de pacientes; há uma redução mais significativa naqueles com DAC.^22^ Como resultado deste estudo, reportou-se que níveis séricos menores de CTRP3 podem ser efetivos na fisiopatologia dessas duas doenças.^22^ Na nossa análise, assim como em outros estudos, observou-se que o nível sérico de CTRP3 foi significativamente menor em pacientes com DM e DAC, em comparação àqueles sem DM. Porém, em nosso estudo, a taxa de pacientes com DM foi maior em comparação a estudos anteriores, e a regulação da glicose no sangue estava relativamente fora do controle. Isso pode ter sido mais efetivo na redução dos níveis séricos de CTRP3.

Nosso estudo tem algumas limitações. Embora os resultados sejam significativos, foram insuficientes em termos do número de pacientes incluídos na análise. Em nossa pesquisa, somente pacientes com DAC estável foram incluídos, e não havia pacientes com SCA. Deveríamos incluir pacientes com SCA. Em nossa análise, embora tenhamos feito as medidas bioquímicas, os níveis de CTRP3 não foram mensurados a partir de amostras de tecido. Achados semelhantes poderiam ser mais significativos ao serem examinados no nível dos miócitos. Em nossa pesquisa, embora a presença de FA tenha sido detectada pelo holter de 72 horas, a carga da FA não foi realizada pelo fato de a amostra ser pequena. O efeito dos medicamentos usados pelos pacientes incluídos em nosso estudo, devido à presença da FA paroxística, não foi avaliado. Poderia ter sido importante avaliar a relação entre a medicação e a presença da FA. Nosso estudo não foi planejado para ter acompanhamento. Um acompanhamento de longo prazo teria trazido resultados mais significativos.

## Conclusão

Os níveis séricos de CTRP3 foram significativamente menores em pacientes com DAC, e relacionados à FA paroxística, o que era comum nesses pacientes. De acordo com nosso estudo e análises prévias investigando o nível de CTRP3 em doenças CV, os níveis séricos de CTRP3 podem ser um parâmetro útil no diagnóstico de pacientes com DAC. Embora a CTRP3 seja muito importante para detectar o desenvolvimento da FA paroxística em pacientes com DAC, nossos achados requerem grupos de pacientes diferentes e precisam ser apoiados por estudos que envolvam mais indivíduos.

### Mensagem principal

Os níveis séricos de CTRP3 foram significativamente mais baixos em pacientes com DAC estável em comparação aos controles saudáveis.

Níveis reduzidos de CTRP3 estiveram relacionados à FA, que é a arritmia mais frequente em pacientes com DAC estável.

### Questões da pesquisa

Outros estudos são necessários para avaliar a significância dos níveis séricos de CTRP3 em pacientes com DAC e fibrilação atrial.

### O que se sabe sobre o tema?

A CTRP3 é um novo membro da família de adipocinas, e está relacionada à inibição do estresse oxidativo, tem efeitos anti-inflamatórios, inibe a apoptose e a fibrose, promove a angiogênese e a inibição da gliconeogênese.

CTRP3 demonstrou reduzir a incidência de doenças cardiovasculares (CV).
