# Supplementary material for: Níveis Reduzidos de CTRP3 em Pacientes com Doença Arterial Coronariana Estável Relacionados à Presença de Fibrilação Atrial Paroxística
Source: Arq Bras Cardiol. 2021 Nov 17;118(1):52–8. [Article in Portuguese] doi: 10.36660/abc.20200669 (PMC8959053; doi:10.36660/abc.20200669)
Supplement: Translation

# Translation: Reduced CTRP3 Levels in Patients with Stable Coronary Artery Disease and Related with the Presence of Paroxysmal Atrial Fibrillation

**Authors:** Arafat Yildirim, Mehmet Kucukosmanoglu, Hilmi Erdem Sumbul, Mevlut Koc

**Affiliations:** 1 Turkey Department of Cardiology, University of Health Sciences - Adana Health Practice and Research Center, Adana - Turkey; 2 Turkey Department of Internal Medicine, University of Health Sciences - Adana Health Practice and Research Center, Adana - Turkey

**Keywords:** Tumor Necrosis Factor Alpha Induced Protein 3, Coronary Artery Disease, Atrial Fibrillation, Risk Factors, Arrhythmias Cardiac

## Abstract

**Background:**

Serum Complement C1q/tumor necrosis factor-related protein-3 (CTRP3) levels and the relationship with atrial fibrillation (AF) in stable coronary artery disease (CAD) are not clearly known.

**Objective:**

The aim of this study was to investigate the change in serum CTRP3 levels and its relationship with paroxysmal AF in stable CAD.

**Method:**

The study included 252 patients with CAD and 50 age-sex matched healthy control subjects. Serum CTRP3 levels were measured in addition to routine anamnesis, physical examination, laboratory and echocardiography examinations. The patients were divided into groups with and without CAD and CAD patients with and without paroxysmal AF. Statistical significance was accepted as p<0.05.

**Results:**

Serum CTRP3 levels were found to be significantly lower in patients with CAD than in the control group (p<0.001). AF was detected in 38 patients (15.08%) in the CAD group. The frequency of hypertension and female gender, hs-CRP, blood urea nitrogen, creatinine levels and left atrial end-diastolic (LAd) diameter were higher (p<0.05 for each one), and CTRP3 levels were lower in patients with AF (p <0.001). In the logistic regression analysis, serum CTRP3 levels and LAd diameters were independently determined the patients with AF (p<0.01 for each one). In this analysis, we found that every 1 ng/mL reduction in CTRP3 levels increased the risk of AF by 10.7%. In the ROC analysis of CTRP3 values for detecting patients with AF, the area under the ROC curve for CTRP3 was 0.971 (0.951–991) and was statistically significant (p<0.001). When the CTRP3 cut-off value was taken as 300 ng/mL, it was found to predict the presence of AF with 87.9% sensitivity and 86.8% specificity.

**Conclusion:**

Serum CTRP3 levels were significantly reduced in patients with stable CAD and decreased CTRP3 levels were closely related to the presence of paroxysmal AF in these patients.

## Introduction

Adipokines are polypeptides that are secreted from the adipose tissue and critical for regulating energy metabolism.^1^ Complement C1q/tumor necrosis factor (TNF) related protein-3 (CTRP3) is a new member of the adipokine family. The main effects of CTRP3 are high glucose-induced oxidative stress inhibition, anti-inflammation, apoptosis inhibition, inhibited fibrosis, promoting angiogenesis and gluconeogenesis inhibition.^2 - 5^ CTRP3, which has been investigated for its metabolic effects, has also been shown to reduce the incidence of cardiovascular (CV) diseases.^6 - 9^

Coronary artery disease (CAD) is the most common cardiovascular disease, whereas atrial fibrillation (AF) is the most common cardiac arrhythmia. The most important and frequent arrhythmia in patients with CAD is atrial fibrillation (AF). Both diseases present risk factors such as hypertension (HT), diabetes mellitus (DM), sleep apnea, obesity and smoking.^10^

CTRP3 levels were investigated in patients with who had acute coronary syndrome (ACS) and stable angina pectoris before.^11^ In this study, serum CTRP3 levels were reported to decrease in patients with ACS and stable angina pectoris, and these results suggest that CTRP3 might be useful for assessing the risk of CAD.^11^ The pathophysiology and mechanism of AF in patients with CAD is complex and multifactorial. However, stretch-induced atrial fibrosis, increased inflammation, vascular remodeling, apoptosis and oxidative stress, hypocontractility, fatty infiltration, ischemia, ion channel dysfunction and Ca2+ instability are closely related to the presence and occurrence of AF.^12^ The fact that decreased CTRP3 levels are closely related to inflammation, fibrosis, apoptosis and oxidative stress has brought us to the hypothesis that AF, which is the most common arrhythmia in patients with CAD, may be associated with reduced CTRP3 levels. To the best of our knowledge, there are no studies which investigate the relationship between serum CTRP3 levels and AF in patients with stable CAD.

Therefore, we aimed to investigate the changes in serum CTRP3 levels in patients with stable CAD and the relationship between CTRP3 levels and the presence of paroxysmal AF in these patients.

## Materials and Methods

### Study population

This cross-sectional study included 252 patients with stable CAD who were admitted to the arrhythmia clinic of our hospital, whose age and sex were similar to 50 healthy controls. Patients were evaluated by two cardiologists for the presence of AF in the 72-hour Holter electrocardiography (ECG) before being included in the study. At least 30 seconds of AF duration is required for the diagnosis of AF.^12^ According to previous studies for comparing CTRP3 means between CAD and control groups with a two-sample t test at a 5% significance level and 80% power, 50 patients are required for each one of the two groups. Based on the knowledge that 17-47 % of CAD patients have AF,^10^ at the beginning of the study, 100 CAD patients were planned to enroll the study in order to analyze at least 30 of them with AF. However, during the study, the number of CAD patients with AF was much lower than the expected. Therefore, the study was carried out with 252 CAD individuals. Patients with acute coronary syndrome, known arrhythmia and anti-arrhythmic drug use, heart failure with reduced ejection fraction, those with a history of liver disease, severe kidney failure, moderate to severe heart valve disease, active thyroid disease, suspected cancer and / or pregnancy, and the ones who did not wish to be included in the study were excluded. The study was conducted according to the recommendations of the Declaration of Helsinki for medical research including human subjects, and the protocol was approved by the institution’s Ethics Committee. Voluntary consent forms were explained in detail to all patients, who were included in the study after providing a written consent.

A detailed history was taken and physical examinations were performed in all patients. Subsequently, the demographic characteristics of all groups were collected; age, gender, history of hypertension (HT), diabetes mellitus (DM), active smoking and hyperlipidemia (HPL) was questioned. Body mass index (BMI) was calculated by measuring weight and height. Laboratory parameters, such as high sensitivity C reactive protein (hs-CRP), glucose, renal and liver functions, N-terminal (NT)-prohormone BNP (NT-proBNP) and lipid values of all patients and healthy controls were measured.

Current treatments continued before the patients were examined for the 72-hour Holter ECG. They were instructed to go on with their routine. A holter monitor programmed for 72 hours with automatic recording and rhythm tracking was used (GE Seer 1000, USA). Patients went home after receiving instructions about the device. After 72 hours, the monitor was removed and the records were reviewed and concluded with the software Mars V8 ambulatory ECG. Detailed information was provided to the patients. The holter results were assessed by two electrophysiologists (AY and MK) for the diagnosis of silent AF, who were unaware that the patients’ clinical and risk factors, as well the patients themselves, were included in the study. As a result of this evaluation, if there was an inconsistent result between the two electrophysiologists (interobserver), the definitive decision was made by a third electrophysiologist in our clinic (MK). In the 12-lead Holter ECG: ı) irregular atrial activity and variability in the length of the atrial cycle (shorter than 200 ms); ıı) irregularity in R-R interval; ııı) absence of recurrent significant P wave; ıv) instead of P waves, fast, irregular, different shapes and sizes of fibrillation waves can be seen; v) finally, irregular and variable ventricular rates were considered as AF.

Blood samples for CTRP3 levels were taken from the patient and control groups at 8 a.m., after a 12-hour fasting period. Venous blood samples were taken and centrifuged at 4000 rpm for at least 10 minutes. We obtained serum samples and kept them stored at -80°C until the analysis. For measuring serum CTRP3 levels, CTRP3 (human) competitive ELİSA (enzyme-linked immunosorbent assay) kits (Adipogen, South Korea, Cat# AG-45A0042EK-KI01) were used. CTRP3 sensitivity was assay within range: 1 ng/mL – 1000 ng/mL, and the results were shown as ng/mL.

All echocardiography examinations were performed on EPIQ 7 (Philips Healthcare, Andover, Massachusetts, USA). The American Echocardiography Society guidelines were used to obtain images. When the patients were monitored and left-sided, a standard parasternal long and short axis was obtained, as well as apical 5, 4 and 2 space chambers, and at least 3 consecutive cycles.^13^ Parasternal long-axis M-mode examination revealed left atrial diastolic (LAd) diameters. LV end-diastolic and systolic (LVd and LVs) volumes and left ventricular ejection fraction (LVEF) were calculated in an echocardiography by using the modified Simpson method from apical 4 and 2 space chambers.^14^

### Statistical analysis

All analyses were performed using the SPSS 22.0 statistical software package (Chicago, IL, USA). The Kolmogorov-Smirnov test was used to analyze the distribution of continuous variables. Continuous variables were expressed as mean ± standard deviation or median - interquartile range. Categorical variables were expressed as numbers and percentage rates. Continuous variables that showed normal distribution were compared using the unpaired sample Student’s t test, whereas the Mann-Whitney U test was used to compare differences between two independent groups when the dependent variable was either ordinal or continuous, but not normally distributed. The chi-square (χ^2^ ) test was used to compare categorical variables. A logistic regression analysis was performed to determine the independent markers among patients with AF. A ROC curve analysis was performed to re-evaluate the CTRP3 levels for detecting patients with AF, and to determine the limit value of CTRP3. The value of the area under the curve was used as a measure of test’s accuracy. Statistical significance was accepted if p <0.05.

## Results

The study data were compared in two groups: patients with stable CAD and the control group. In addition, patients with CAD were grouped and compared as patients with and without paroxysmal AF. Of the total number of 302 individuals, 252 patients with stable CAD (female, n=85, 33.7%; mean age 61.6 ± 11.4) and 50 healthy controls (female, n=20, 40.0%; mean age: 60.4 ± 9.2) were included in the study. Of the CAD patients included in the study, 161 (63.9%) had HT, 84 (33.3%) had DM, 86 (34.1%) had HPL and 69 (27.4%) were active smokers. AF was detected in 38 (15.07%) patients with stable CAD.

Demographic characteristics and laboratory findings of all participants are summarized in Table 1 . When the demographic characteristics of the patients and control subjects were compared, age and gender were found to be similar (61.6 ± 11.4 vs. 60.4 ± 9.2; p=0.514 and 33.7% vs. 40.0%, p=0.224, respectively). When compared with the control subjects, serum glucose, total cholesterol and low-density lipoprotein (LDL) cholesterol levels were found to be significantly higher in patients with stable CAD (146 ± 75 vs. 95 ±12; p<0.001, 184 ± 42 vs. 152 ± 36; p=0.012, 120 ± 34 vs. 100 ± 25; p=0.020, respectively). Serum CTRP3 levels were statistically lower, whereas hs-CRP levels were higher in patients with stable CAD (331 ± 46 vs. 432 ± 46; p<0.001 and 1.09 (0.95 – 1.31), 0.89 (0.76 – 1.01); p=0.030, respectively). Other laboratory data were similar between the two groups. When echocardiography parameters were compared, LAd dimensions were significantly higher (43 ± 4.6 vs. 38 ± 4.1; p=0.012), and LVEF was significantly lower (52 ± 6.9 vs. 61 ± 4.5; p=0.019) in patients with stable CAD.

**Table 1:** – Demographic and laboratory findings of patients with coronary artery disease and healthy controls

Variable	Patients with CAD n=252	Healthy Controls n=50	p
Age (years)	61.6 ± 11.4	60.4 ± 9.2	0.514
Gender (female), n (%)	85 (33.7)	20 (40.0)	0.224
BMI (kg/m^2^)	25.4 ± 2.23	25.9 ± 1.30	0.122
Glucose (mg/dL)	146 ± 75	95 ± 12	<0.001
BUN (mg/dL)	36.2 ± 15.6	33.1 ± 14.5	0.192
Creatinine (mg/dL)	0.90 (0.75 – 0.99)	0.80 (0.70 – 0.92)	0.334
Total cholesterol (mg/dL)	184 ± 42	152 ± 36	**0.012**
LDL cholesterol (mg/dL)	120 ± 34	100 ± 25	**0.020**
HDL cholesterol (mg/dL)	40 ± 12	42 ± 9.8	0.856
Triglycerides (mg/dL)	149 (130 – 175)	151 (125 – 186)	0.956
hs-CRP (mg/dL)	1.09 (0.95 – 1.31)	0.89 (0.76 – 1.01)	**0.030**
CTRP3 (ng/mL)	331 ± 46	432 ± 46	**<0.001**
LVd volume (mL)	102 ± 19	95 ± 11	0.102
LVs volume (mL)	46 ± 14	41 ± 10	0.112
LAd diameter (mm)	43 ± 4.6	38 ± 4.1	**0.012**
LVEF (%)	52 ± 6.9	61 ± 4.5	**0.019**

*The values were shown as mean ± standard deviation, median - interquartile range or n (%), BMI: body mass index; BUN: blood urea nitrogen; CAD: coronary artery disease; CTRP3: complement C1q/tumor necrosis factor (TNF)-related protein-3; HDL: high density lipoprotein; hs-CRP: high sensitivity C reactive protein; LDL: low density lipoprotein; LVEF: left ventricular ejection fraction; LVd: left ventricular diastolic; LVs: left ventricular systolic; LAd: left atrial diastolic.*

Demographic characteristics and laboratory findings of patients with stable CAD according to presence of AF are summarized in Table 2 . Demographic data of the patients with and without paroxysmal AF, frequency of the female gender, age and HT were statistically higher in patients with AF (50.0% vs. 30.8%; p= 0.019, 66.8 ± 10.7 vs. 60.3 ± 11.2; p=0.001, 78.9% vs. 61.2%; p=0.025, respectively). When compared with the stable CAD patients without paroxysmal AF, serum blood urea nitrogen and creatinine levels [44.5 ± 21.9 vs. 34.8 ± 13.8; p= 0.012, 1.20 (0.90 – 1.45) vs. 0.85 (0.70 – 1.35); p=0.004, respectively] were significantly higher in patients with stable CAD and paroxysmal AF. Besides, serum CTRP3 levels were statistically lower, whereas hs-CRP levels were higher in patients with stable CAD and paroxysmal AF [262 ± 27 vs. 343 ± 38; p=<0.001, 3.15 (2.22 – 3.95) vs. 0.80 (0.65 -1.02); p<0.001, respectively]. Other laboratory data were similar between the two groups. When echocardiography parameters were compared, the LAd diameter was significantly higher (46 ± 5.4 vs. 41 ± 4.2; p=0.002) and LVEF was significantly lower (49 ± 4.9 vs. 53 ± 6.1; p=0.049) in patients with stable CAD and paroxysmal AF.

**Table 2:** – Demographic and laboratory findings of CAD patients according to the presence of atrial fibrillation.

Variable	Paroxysmal Atrial fibrillation (+) n=38	Paroxysmal Atrial fibrillation (-) n=214	p
Age (years)	66.8 ± 10.7	60.3 ± 11.2	**0.001**
Gender (female), n (%)	19 (50.0)	66 (30.8)	**0.019**
Hypertension, n (%)	30 (78.9)	131 (61.2)	**0.025**
Diabetes mellitus, n (%)	15 (39.5)	69 (32.2)	0.245
Current smoker, n (%)	4 (10.5)	65 (30.4)	0.224
Hyperlipidemia, n (%)	13 (34.2)	73 (34.1)	0.224
BMI (kg/m^2^)	25.3 ± 2.18	25.5 ± 2.24	0.576
Glucose (mg/dL)	160 ± 65	144 ± 78	0.207
BUN (mg/dL)	44.5 ± 21.9	34.8 ± 13.8	0.012
Creatinine (mg/dL)	1.20 (0.90 – 1.45)	0.85 (0.70 – 1.35)	**0.004**
Total cholesterol (mg/dL)	179 ± 42	184 ± 42	0.518
LDL cholesterol (mg/dL)	116 ± 33	121± 34	0.380
HDL cholesterol (mg/dL)	41 ± 13	40 ± 12	0.823
Triglycerides (mg/dL)	140 (115 – 165)	150 (110 -182)	0.663
hs-CRP (mg/dL)	3.15 (2.22 – 3.95)	0.80 (0.65 -1.02)	**<0.001**
CTRP3 (ng/mL)	262 ± 27	343 ± 38	**<0.001**
LVd volume (mL)	104 ± 22	96 ± 18	0.305
LVs volume (mL)	52 ± 15	48 ± 11	0.456
LAd diameter (mm)	46 ± 5.4	41 ± 4.2	**0.002**
LVEF (%)	49 ± 4.9	53 ± 6.1	**0.049**

*The values were shown as mean ± standard deviation, median - interquartile range or n (%),BMI: body mass index; BUN: blood urea nitrogen; CAD: coronary artery disease; CTRP3: complement C1q/tumor necrosis factor (TNF)-related protein-3; HDL: high density lipoprotein; hs-CRP: high sensitivity C reactive protein; LAd: left atrial diastolic; LDL: low density lipoprotein; LVEF: left ventricular ejection fraction; LVd: left ventricular diameter diastolic; LVs: left ventricular diameter systolic.*

Demographic, clinical and laboratory parameters associated with paroxysmal AF in the univariate analysis were evaluated by multivariate logistic regression. Serum CTRP3 levels (OR: 0.893; 95%CI, 0.856–0.931; p<0.001) and LAd dimension values (OR: 1.160; 95%CI, 1,101–1,229; p=0.003) were found to independently determine the patients with paroxysmal AF ( Table 3 ). In this analysis, every 1 ng/mL reduction in the CTRP3 level was found to increase the probability of having paroxysmal AF by 10.7%.

**Table 3:** – Variable regression analysis for the detection of CAD patients with atrial fibrillation

Variable	OR	95%CI	p
LAd diameter (mm)	1.160	1.101 – 1.229	**0.003**
CTRP3 (ng/mL)	0.893	0.856 – 0.931	**<0.001**

*CTRP3: complement C1q/tumor necrosis factor (TNF)-related protein-3; LAd: left atrial diastolic; OR: odds ratio; CI: confidence interval.*

In the ROC analysis of CTRP3 values for detecting patients with paroxysmal AF, the area under the ROC curve for CTRP3 was 0.971 (95%CI: 0.951–0.991), and it was statistically significant (p<0.001 and Figure 1 ). When the cutoff point of CTRP3 was considered as 300ng/mL, it was found to predict AF with 87.9% sensitivity and 86.8% specificity.

**Figure 1:** – Receiver operating characteristic curves of CTRP values in predicting patients with paroxysmal AF.

## Discussion

The most important finding of our study is that serum CTRP3 levels were significantly lower in patients with stable CAD compared to healthy controls, and reduced CTRP3 levels were closely related to paroxysmal AF, which is the most frequent arrhythmia in patients with stable CAD. In the literature, only one study reported that serum CTRP3 levels decreased in patients with stable angina pectoris and ACS; however, our study is about the importance of serum CTRP3 levels in predicting the AF that may present itself in these patients. When the limit value for serum CTRP3 level is considered as 300 ng/mL, it predicts the risk of having paroxysmal AF with acceptable sensitivity and specificity. For this reason, our study provides important data to the literature.

In the studies about the effects of CTRP3 on the cardiovascular system: it i) reduces oxidative stress, ii) inhibits apoptosis, iii) has anti-inflammatory and anti-atherogenic effects, iv) reduces the development of fibrosis, v) inhibits gluconeogenesis and, as a result of all these effects, CTRP3 reduces the chances of CV diseases.^2^ Considering its current effects, it was found that CTRP3 can, in physiopathological terms, protect and improve the clinical features of patients with CAD. Serum CTRP3 levels were investigated in patients with heart failure with reduced ejection fraction (HFrEF), acute coronary syndrome, stable angina pectoris and acute aortic dissection; and serum CTRP3 levels were reported to be significantly lower.^6 , 11 , 17^ In *in vivo* studies on mice, CTRP3 has been shown to improve cardiac contractile functions with anti-apoptotic and pro-angiogenic effects after myocardial ischemia.^18^ Similarly, in patients with acute coronary syndrome and stable angina, serum CTRP3 levels were reported to be significantly lower than in healthy controls.^11^ CTRP3 may also have had these positive effects with anti-atherogenic and anti-inflammatory effects.^15^

The most important and frequent arrhythmia in patients with CAD is AF. Both diseases present risk factors such as hypertension, diabetes mellitus, sleep apnea, obesity and smoking. The prevalence of CAD in patients with AF is from 17 to 46.5%, whereas the prevalence of AF among patients with CAD is low and, estimated to range from 0.2 to 5%.^10^ Moreover, inflammation plays a causative role in both diseases. It is not easy to explain the AF pathogenesis in patients with CAD by a mechanism. Activation of fibroblasts, enhanced connective tissue deposition, and fibrosis are the hallmarks of this process.^12^ In addition, atrial fatty infiltration, inflammatory infiltrates, myocytes hypertrophy, necrosis, and amyloidosis are found in patients with AF and concomitant conditions predisposing to AF.

The fact that the CTRP3 level has a positive effect on essential mechanisms that are effective in the pathophysiology of AF led us to consider that there may be a relationship between CTRP3 levels and AF. As a result, in our study, the serum CTRP3 level was found to be significantly lower in the patient group with AF due to CAD compared to the patient group without AF. Therefore, we aimed to investigate the changes in serum CTRP3 levels in patients with stable CAD and the relationship between CTRP3 levels and the presence of paroxysmal AF in these patients.

Many previous studies have reported that dysmetabolic risk factors, such as obesity, increased waist circumference, blood pressure, fasting serum glucose and insulin resistance are negatively associated with serum CTRP-3 levels.^19 - 21^ A similar finding was demonstrated by the significantly lower serum CTRP-3 level in patients with DM.^22^ CAD and DM are closely related diseases, and it has been shown that serum CTRP-3 levels are reduced in both patient groups; there is a more significant decrease in those with CAD.^22^ As a result of this study, it was reported that a reduced serum CTRP-3 level may be effective in the pathophysiology of these two diseases.^22^ In our analysis, similarly to previous studies, it was observed that the serum CTRP-3 level was significantly lower in patients with DM together with CAD compared to those without DM. However, in our study, the rate of patients with DM was higher in comparison to previous studies, and blood glucose regulation was relatively not under control. This may have been more effective in the reduction of serum CTRP-3 levels.

Our study has some important limitations. Although the results were significant, they were insufficient in terms of the number of patients included in the study. In our analysis, only patients with stable CAD were included, and there were no patients with ACS. We should include patients with ACS in our study. In our analysis, although biochemical measurements were taken, CTRP3 levels were not measured from tissue samples. Similar findings could be more meaningful to examine at the level of myocytes. In our research, although the presence of AF was detected by 72-hour Holter ECG recordings, the AF burden evaluation was not performed because the sample was small. The effect of the drugs used by the patients included in our study due to the presence of paroxysmal AF was not evaluated. It could have been more meaningful if the relationship between medication and the presence of AF had been evaluated. Our study was not planned to be a follow-up study. Long-term follow-up would have brought more meaningful results.

## Conclusion

Serum CTRP levels were significantly reduced in patients with CAD and closely related to paroxysmal AF, which was common in these patients. According to our study and previous analyses investigating the level of CTRP3 levels in CV diseases, serum CTRP3 levels may be a useful parameter in the diagnosis of patients with CAD. Although CTRP3 is found to be very important in detecting paroxysmal AF development in CAD patients, our current findings require different patient groups and need to be supported by studies involving more patients.

### Main message

The serum CTRP3 levels were significantly lower in patients with stable coronary artery disease compared to healthy controls.

Reduced CTRP3 levels were closely related to AF, which is the most frequent arrhythmia in patients with stable CAD.

### Current research questions

Further studies are required to evaluate the significance of serum CTRP3 levels in patients with CAD and atrial fibrillation.

### What is already known on the subject?

CTRP3 is a new member of the adipokine family and is related to oxidative stress inhibition, anti-inflammation, apoptosis inhibition, inhibited fibrosis, promoting angiogenesis and gluconeogenesis inhibition.

CTRP3 has been shown to reduce the incidence of cardiovascular (CV) diseases.
